# Value of Shape and Texture Features from ^18^F-FDG PET/CT to Discriminate between Benign and Malignant Solitary Pulmonary Nodules: An Experimental Evaluation

**DOI:** 10.3390/diagnostics10090696

**Published:** 2020-09-15

**Authors:** Barbara Palumbo, Francesco Bianconi, Isabella Palumbo, Mario Luca Fravolini, Matteo Minestrini, Susanna Nuvoli, Maria Lina Stazza, Maria Rondini, Angela Spanu

**Affiliations:** 1 Section of Nuclear Medicine and Health Physics, Department of Surgical and Biomedical Sciences, Università degli Studi di Perugia, Piazza Lucio Severi 1, 06132 Perugia, Italy; barbara.palumbo@unipg.it (B.P.); matteo.minestrini@ospedale.perugia.it (M.M.); 2 Department of Engineering, Università degli Studi di Perugia, Via Goffredo Duranti 93, 06135 Perugia, Italy; mario.fravolini@unipg.it; 3 Section of Radiation Oncology, Department of Surgical and Biomedical Sciences, Università degli Studi di Perugia, Piazza Lucio Severi 1, 06132 Perugia, Italy; isabella.palumbo@unipg.it; 4 Unit of Nuclear Medicine, Department of Medical, Surgical and Experimental Sciences, Università degli Studi di Sassari, Viale San Pietro 8, 07100 Sassari, Italy; snuvoli@uniss.it (S.N.); marialinastazza@yahoo.it (M.L.S.); maria.rondini01@ateneopv.it (M.R.); aspanu@uniss.it (A.S.)

**Keywords:** solitary pulmonary nodule, shape, texture, radiomics

## Abstract

In this paper, we investigate the role of shape and texture features from ${}^{18}$F-FDG PET/CT to discriminate between benign and malignant solitary pulmonary nodules. To this end, we retrospectively evaluated cross-sectional data from 111 patients (64 males, 47 females, age = 67.5 ± 11.0) all with histologically confirmed benign (n=39) or malignant (n=72) solitary pulmonary nodules. Eighteen three-dimensional imaging features, including conventional, texture, and shape features from PET and CT were tested for significant differences (Wilcoxon-Mann-Withney) between the benign and malignant groups. Prediction models based on different feature sets and three classification strategies (Classification Tree, k-Nearest Neighbours, and Naïve Bayes) were also evaluated to assess the potential benefit of shape and texture features compared with conventional imaging features alone. Eight features from CT and 15 from PET were significantly different between the benign and malignant groups. Adding shape and texture features increased the performance of both the CT-based and PET-based prediction models with overall accuracy gain being 3.4–11.2 pp and 2.2–10.2 pp, respectively. In conclusion, we found that shape and texture features from ${}^{18}$F-FDG PET/CT can lead to a better discrimination between benign and malignant lung nodules by increasing the accuracy of the prediction models by an appreciable margin.

## 1. Introduction

A solitary pulmonary nodule (SPN) is usually defined as an “approximately round lesion that is less than 3 cm in diameter and that is completely surrounded by pulmonary parenchyma, without other abnormalities” [1]. With the widespread availability of chest CT and the improving capabilities of the imaging devices, the incidence of SPN has been rising constantly in recent years [2,3]. In the United States, the estimated detection rate (defined as at least one positive CT scan) increased from 3.9 per 1000 person-years in 2006 to 6.4 in 2012, of which the fraction of those who received a diagnosis of lung cancer within two years from the transcript was 5.2% [4]. Other works have reported estimates about the prevalence and malignancy rate of SPN between 2% and 69% [2,5,6] and between 7% and 40% [3,7,8], respectively.

Differential diagnosis of SPN includes benign aetiologies, such as pneumonia, fungi infection, tuberculosis, and hamartoma, as well as malignant ones—most commonly primary lung cancer, distant (metastatic) lesions, or lymphoma [7,9]. Assessment of the malignancy risk involves the evaluation of clinical and radiographic variables. Age, history of smoking, and exposure to carcinogenic agents are well-known risk factors, although their absence does not preclude malignancy [9]. Radiographic findings that are suggestive of benignity or malignancy include size, density, stability over time, margin appearance, wall thickness, and the presence of cavitation and calcification [9,10,11]. Clinical management of patients with SPN is determined on the basis of the risk assessed, and may involve routine CT follow-ups, functional imaging, and/or tissue sampling [7,9,12].

In recent years, quantitative extraction of imaging features from medical scans (“radiomics” [13,14,15]) has attracted widespread interest as a possible means to discriminate between benign vs. malignant SPN [16]. The rationale behind radiomics is to leverage on that fraction of image information which may have clinical relevance but go unnoticed to the human eye [17]. Radiomics also enables full-field analysis of the region of interest, while biopsies only capture a small portion of the lesion [18]. Several studies have proposed predictive models based on a range of combinations of radiomics features from CT, with overall reported accuracy between 70% and 95% [19,20,21,22,23,24,25,26]. Uptake parameters from ${}^{18}$F fluodeoxyglucose (FDG) Positron Emission Tomography (PET henceforth) have also shown good diagnostic performance (accuracy between 65% and 91% in [3,8,27,28,29,30]) with potential improvements coming from the characterisation of uptake heterogeneity [31,32]. In a recent meta-analysis, Jia et al. [33] concluded that CT and PET/CT have both moderate-to-high diagnostic value in patients with SPN, with no significant differences between the two modalities.

Few studies, however, have addressed the problem of quantifying the gain that shape and texture features from CT and/or PET/CT may provide compared with conventional imaging features alone. Among them, Wu et al. [25] reported that adding texture features from CT to clinical and semantic variables could lead to a 0.03 increase of the area under the curve (AUC), whereas Balagurunathan et al. [23] determined that a combination of size, shape, and texture features could increment the AUC from 0.87 to 0.90 compared with a model based on longest diameter and volume of the nodule only.

In this work, we investigated whether shape and texture features from PET/CT could lead to a better discrimination between benign and malignant SPN compared with standard imaging features alone—that is, lesion size, density, and radiotracer uptake. To this end, we retrospectively evaluated the baseline PET/CT scans of 111 patients—all with histologically confirmed benign or malignant lesions—who had a positive transcript for SPN. The statistical analysis was carried out in two steps: we first screened the features for significance against benignity vs. malignancy, then used the significant features to build prediction models of different complexity.

## 2. Materials and Methods

### 2.1. Study Population

**Figure 1:**
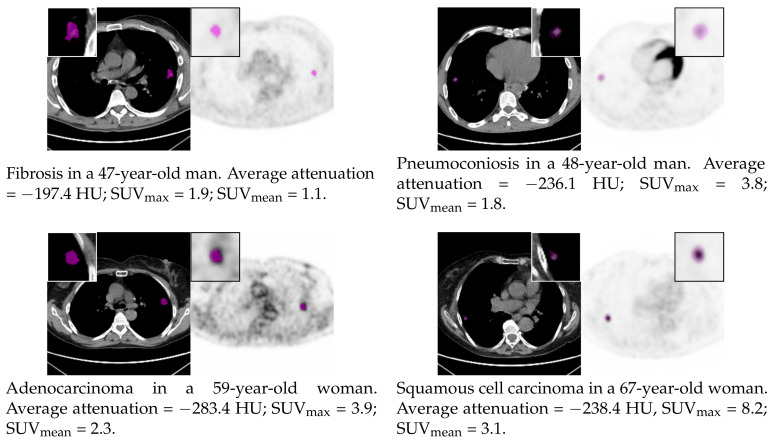
Sample CT and PET scans showing benign (first row) and malignant (second row) pulmonary nodules. Magnified (2×) views of the manually segmented lesions are shown in the insets; basic CT and PET parameters are also reported.

We retrospectively evaluated a cohort of 111 patients (details of the study population in Table 1; commented sample scans in Figure 1) who underwent PET/CT examination for suspicious lung nodules at the Unit of Nuclear Medicine of the Università degli Studi di Sassari, Sassari, Italy, between November 2014 and May 2019. The inclusion criteria were: (1) presence of a clearly identifiable solid nodule at CT above 5 mm and up to 40 mm in maximum axial diameter (same criterion adopted in [29]), (2) no previous surgery, chemotherapy, and/or radiotherapy for the inspected lesion, and (3) histologically confirmed malignancy or benignity. The maximum axial diameter (volume) of the benign lesions at CT ranged between 7.4 mm and 35.8 mm (165.0 mm${}^{3}$ and 11,222.2 mm${}^{3}$), and that of the malignant ones between 10.0 mm and 38.1 mm (421.7 mm${}^{3}$ and 18440.9 mm${}^{3}$). Known risk factors in the patient series were: history of tuberculosis (prevalence in the study population = 5.4%), fibrosis (8.1%), pulmonary emphysema (8.1%), chronic obstructive pulmonary disease (17.1%), rheumatoid arthritis (5.4%), previous malignancy (14.4%), current smoking (23.4%), former smoking (25.2%), exposure to asbestos (5.4%), and exposure to other chemicals and/or agents (7.2%). The standard of reference for all the lesions was histological evaluation after bronchoscopy and/or surgical resection.

All patients gave written informed consent as part of the examination routine, and their data were treated according to the local privacy rules and regulations. Request for an ethical standard was waived due to the retrospective nature of the study.

### 2.2. Acquisition Protocol

All the patients fasted for at least 6 h and were checked for blood glucose level <150 mg/dL before examination. Afterwards, they were administered an intravenous bolus of 3.75 Mbq/Kg, and whole-body image acquisition started 60 min thereafter. PET images of size 256 px × 256 px (slice thickness 3.27 mm, in-plane pixel spacing ≈2.73 mm in both directions) were acquired in helicoidal mode and reconstructed via an iterative reconstruction algorithm (GE-VPFXS). Computed tomography scans for attenuation correction were also acquired in helicoidal mode with tube voltage 120 kVp and automatically adjusted tube current. The other CT settings were: slice thickness 3.75 mm, spacing between slices 3.27 mm, in-plane inter-voxel spacing ≈1.37 mm in both directions, and image size 512 px × 512 px. All scans were performed on a Discovery 710 PET/CT system (GE Healthcare, Chicago, IL, USA).

### 2.3. Lesion Segmentation

Delineation of the region of interest (ROI) was performed by consensus by a panel of one radiation oncologist (I.P., >15 yr experience) and one nuclear medicine specialist (B.P., >20 yr experience) on the cross-platform, open-access software LIFEx 5.10 [34,35]. The process was carried out manually and separately for the CT and PET scans with no automatic transposition of the resulting ROI from PET to CT, or vice versa.

### 2.4. Extraction of Radiomics Features

From both the PET and CT images, we extracted five conventional features, four first-order statistics, six second-order statistics, and three shape features, as detailed in Table 2. For the features that require binning (marked with an asterisk in the Table), we used absolute quantisation into 256 levels over the following fixed-width intervals: [−1230HU,235HU] for CT (“lung” window [36], giving a bin width of ≈5.7 HU) and [0SUV,40SUV] for PET (bin width ≈0.16 SUV). Features from GLCM and NGTDM were computed using 26-connectivity and inter-voxel distance δ=1. No further pre-processing, like image normalisation, spatial resampling, or filtering was applied to the CT or PET images before feature extraction. Post-processing involved feature scaling through min–max normalisation ([37], [Equation 3.8]). The calculations were performed through the open-source Python package Pyradiomics 2.2.0, to the documentation of which we refer the reader for details about feature definition, implementation, and mathematical formulae [38,39]. Preliminary conversion from DICOM (.dcm) format (in which the original scans were available) to Pyradiomics-readable NRRD format (.nrrd) was carried out on the open-source 3D Slicer platform 4.10.2 [40,41].

### 2.5. Statistical Analysis

We carried out a statistical analysis in two steps. In the first, we tested each of the CT and PET features for significant differences between the benign and malignant groups. In the second, we determined whether shape and texture features could lead to a better prediction of malignant and benign nodules compared with conventional imaging features alone.

#### 2.5.1. Significant Features

Hypothesis testing was based on non-parametric Wilcoxon-Mann-Witney test [42], the null hypothesis being that the distribution of each feature was the same in the benign and malignant groups. We set a significance level α=0.05 and applied Bonferroni’s correction to protect from type-I error due to multiple tests [43]. Since we tested 18 features and two image modalities, differences were deemed significant if the *p*-value <α/(18×2).

#### 2.5.2. Assessment of Prediction Accuracy

**Figure 2:**
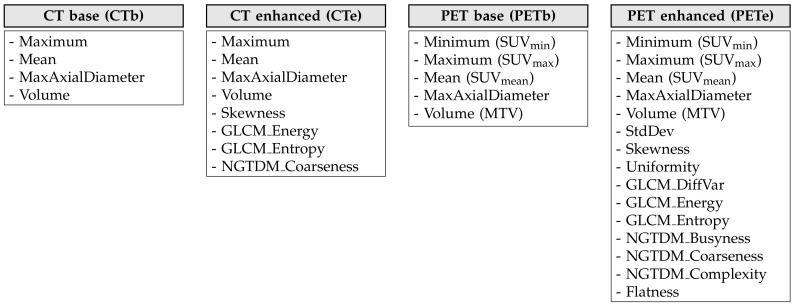
The four feature sets used to build the prediction models.

To discriminate between benign and malignant nodules, we considered four feature combinations: two “base”’ sets made up of conventional imaging features (“CT base”, “PET base”), and two “enhanced” versions (“CT enhanced”, “PET enhanced”) in which the conventional features were complemented with texture and shape features. For all the sets, we only took into account those features that were significantly different between the benign and malignant groups as results from Table 3 and Table 4 (see also Figure 2 for a round-up diagram).

To assess the ability of the above feature sets to discriminate between benign vs. malignant nodules, we built, for each set, three prediction models based on the following classification strategies: Classification Tree (“ClT”), k-Nearest Neighbours (KNN), and Naïve Bayes (“NB”). We used k=1 and $L_{1}$ (“cityblock”) distance for KNN, a Gaussian kernel for NB, and the default settings available in scikit.learn [44] for ClT.

For all the combination feature sets/classifiers, the ability to discriminate benign vs. malignant lesions was estimated following the same protocol described in [23]—that is, we used 80% of the samples of each class (train set) to train the prediction model, and the remaining 20% (test set) to estimate its accuracy. For a stable estimation, we repeated the random split into training and test sets 200 times and averaged the results. For each classifier, we checked for statistically significant differences in accuracy, sensitivity, and specificity between the “base” and “enhanced” sets. To this end, we again used Wilcoxon-Mann-Witney test (α=0.05) over the performance figures resulting from the 200 splits.

## 3. Results

Table 3 and Table 4 compare the values of the radiomics features in the benign (“N”) and malignant (“P”) groups. The CT features (Table 3) indicate that nodule size (volume, maximum diameter) and tissue density (mean, maximum) were significantly higher in the malignant group. The density distribution was also significantly more left-skewed in the malignant lesions than in the benign ones. The second-order statistics show that GLCM energy and entropy were, respectively, significantly lower and higher in the malignant group—both results indicating higher heterogeneity of the malignant lesions compared with the benign ones. This is also consistent with NGTDM coarseness, which was significantly higher in the benign nodules (more uniform texture) than in the malignant ones.

The radiomics features from PET showed that radiotracer uptake (SUV${}_{\min}$, SUV${}_{\max}$, and SUV${}_{\mathrm{mean}}$) was higher in the malignant group, as one would reasonably expect. First-order statistics, like standard deviation and uniformity, were also different—higher and lower in the malignant group, respectively—both indicating that malignant lesions had a higher degree of heterogeneity/disorder. This trend was consistent with the second-order features, all of which were significantly different between the two groups; specifically, GLCM diffvar, GLCM entropy, and NGTDM complexity were higher in the malignant group, whereas GLCM energy, NGTDM busyness, and NGTDM coarseness were lower.

As for the shape features, only the flatness computed on PET was significantly different between the two groups and higher in the malignant one.

Table 5 reports the estimated performance (accuracy, sensitivity, and specificity) of the different combination feature sets/classifiers. As can be seen, adding texture features to the basic models always increased accuracy (3.4–11.2 pp gain for the CT-based models, 2.2–10.0 pp for the PET-based) and sensitivity (3.4–12.8 pp for the CT-based models, 3.4–17.7 pp for the PET-based) by an appreciable margin. Specificity also increased for all the CT-based models (6.0–8.3 pp), but in one case, decreased for the PET-based models (−3.9–4.5 pp).

## 4. Discussion

The recent literature has consistently emphasized the potentially useful role that shape and texture features from PET/CT could play in the characterisation of suspicious pulmonary nodules. Still, the validity and implications of these results need to be understood better before SPN radiomics can be translated into clinical practice [13,16].

One crucial point is to quantify the actual benefit that radiomics features can provide beyond conventional imaging parameters alone. In our experiments, shape and texture features were able to improve the overall accuracy of the prediction models by 2.2–11.2 pp. The increase in CT accuracy found here is comparable, in magnitude, with that reported by Wu et al. [25] and Balagurunathan et al. [23]. However, our overall accuracy was, in absolute terms, lower than that reported in the above references, most probably because those results were obtained with standard CT scans, ours with low-dose ones. For a comparison, Miwa et al. [32] obtained 62.9% accuracy with CT features extracted from low-dose scans, a result very much in line with our figures.

Texture analysis at CT (Table 3) showed that malignant lesions had higher GLCM entropy, lower GLCM energy, and lower NGTDM coarseness than benign lesions, all indicating that malignant nodules had a higher degree of heterogeneity than benign ones. This finding is in agreement with the results reported by Zhao et al. [45] (though their study was two-dimensional), and in general, seems to confirm image heterogeneity at CT as an indicator of malignancy, as previous studies have suggested [46,47]. Skewness was also different between the benign and malignant groups, with the former exhibiting more negatively skewed distributions (excess of high vales) than the benign ones. The meaning and potential implications of this finding, however, are unclear and should be further evaluated in future studies.

It has long been speculated that some characteristics of pulmonary nodules at CT could help differentiate between benign and malignant lung nodules. In particular, irregular, “spiculated” lesions are usually considered more likely to be malignant than round ones with well-defined, smooth margins [1,11]. Although this hypothesis has found some confirmation in previous studies [20,48], we did not find further support for it in our investigation: among the shape features considered here (i.e., sphericity, elongation, and flatness), no one was significantly different between the benign and malignant groups.

The role of tissue density has also been a subject of debate in a number of studies and never clarified completely. Fat density (attenuation values between −120 HU and −40 HU) is considered a possible indicator of hamartomas, and attenuation values greater than 200 HU of calcification [10,11]. In [49], however, the authors concluded that baseline nodule density could not be used to discriminate between benign and malignant SPN. Here, we found that tissue density was significantly higher in the malignant group, although the intra-class variation was considerable (mean value −243.2 ± 126.9 HU and −155.2 ± 112.3 HU, respectively, for the benign and malignant nodules).

Fifteen PET features (Table 4) were significantly different between the benign and malignant groups—that is, nearly twice as many as the CT features. Besides, the models based on PET features in general outperformed those based on CT features, which is in agreement with previously published results [29,32,50]. This is interesting, since it suggests that assessing the heterogeneity of the radiotracer uptake via texture features from PET (as was discussed, for instance, in [5,6]) can be at least as important as doing the same on tissue density via texture features from CT, although the literature has mostly focused on the latter so far.

All the first-order and second-order statistics indicate that malignant lesions had higher uptake heterogeneity than the benign ones. In particular, standard deviation, GLCM diffvar, GLCM entropy, and GLCM complexity were higher for the positive group, whereas uniformity, GLCM energy, and GLCM coarseness were lower. NGTDM busyness was lower in the positive group, which is consistent with the larger uptake range in the malignant lesions, and is in agreement with the results reported in [28]. The uptake distribution in the malignant group was more positively skewed (excess of low vales) than in the benign one, although this finding is not readily interpretable and the potential implications of it are unclear.

Interestingly, the results showed some dependence on the classification model used. Whereas adding shape and texture features always increased the overall prediction accuracy, we noticed that with the Naïve Bayes classifier and CT features, there was a negative effect on sensitivity, which dropped by 3.0 pp.

## 5. Conclusions

We found that shape and texture features from ${}^{18}$F-FDG PET/CT can provide added value in the discrimination between benign and malignant lung nodules compared with conventional imaging features alone. This work is not exempt from limitations—among them are the relatively contained sample size and the retrospective nature. The findings should be further confirmed in larger, ideally prospective studies. By design, we also focused on evaluating the potential benefit of radiomics features compared with conventional imaging features; therefore, the effects of other potentially relevant data, such as nodule location, previous exposure to risk factors, age and/or gender were not investigated here.

**Ethical Statement:** This retrospective study was carried out following the ethical standards of the institutional research committee and with the 1964 Helsinki declaration and its later amendments or ethical standards. The requirement to obtain informed consent was waived as this was a retrospective study. All patient data were treated following the local privacy regulations.

## Figures and Tables

**Table 1:** Characteristics of the patient series.

Attribute [Data Format]	Value
*Demographics*
Age [mean ± SD]	67.5 ± 11.0
Female [*N* (%)]	47 (42.3)
Male [*N* (%)]	64 (57.7)
*Histology*
Benign [*N* (%)]	39 (35.1)
Anthracosis [*N* (%)]	1 (0.9)
Fibrosis [*N* (%)]	10 (9.0)
Inflammation [*N* (%)]	26 (23.4)
Metaplasia [*N* (%)]	1 (0.9)
Pneumoconiosis [*N* (%)]	1 (0.9)
Malignant [*N* (%)]	72 (64.9)
Adenocarcinoma [*N* (%)]	46 (41.4)
Atypical carcinoid (NSCLC) [*N* (%)]	1 (0.9)
Metastasis [*N* (%)]	1 (0.9)
Neuroendocrine tumour [*N* (%)]	1 (0.9)
Small-cell lung cancer [*N* (%)]	2 (1.8)
Spinocellular carcinoma [*N* (%)]	4 (3.6)
Squamous cell carcinoma [*N* (%)]	9 (8.1)
Unspecified [*N* (%)]	8 (7.2)

**Table 2:** Summary table of the radiomics features considered in the study. Key to abbreviations: ‘GLCM’ = Grey-Level Co-occurrence Matrix, ‘NGTDM’ = Neighbouring Gray-Tone Difference Matrix. Features that require binning are marked with an asterisk.

Group/Name	Definition/Interpretation
*Conventional features*	
Maximum	Maximum value (corresponds to SUV${}_{\max}$ when computed on PET).
Minimum	Minimum value (corresponds to SUV${}_{\min}$ when computed on PET).
Mean	Mean (average) value (corresponds to SUV${}_{\mathrm{mean}}$ when computed on PET).
MaxAxialDiam	Largest pairwise Euclidean distance between the surface mesh of the lesion in the axial plane.
Volume	The volume of the lesion computed by summing up the volume of each voxel in the ROI. Usually referred to as Metabolic Tumour Volume (MTV) when computed on PET.
*First-order statistics*	
StdDev	Standard deviation, a measure of the data variability. Low values indicate the data are close to the mean; high values that they are spread over a large range.
Skewness	A measure of the distribution’s departure from symmetry. Negative values indicate a left-skewed distribution (peak toward the right, left tail longer), positive values a right-skewed distribution (peak toward the left, right tail longer).
Kurtosis	A measure of the tailedness of the data compared to that of a normal distribution. High values indicate strongly-tailed data (presence of outliers), low values weakly-tailed data (absence of outliers).
Uniformity *	Sum of the squared probabilities of the intensity levels after binning. Measures the distribution’s departure from uniformity. High values indicate that few intensity levels are more likely to occur than the other levels, low values that all the intensity levels are equally likely to occur.
*Second-order statistics*	
GLCM_DiffVar *	A measure of heterogeneity in which the occurrence probability of pairs of voxels whose intensity difference is far from the average is weighted more than that of pairs of pixels whose difference is close to the average.
GLCM_Energy *	The equivalent to first-order Uniformity (see above) for the joint distribution of pairs of voxel intensities. High values indicate that few pairs of intensity levels are more likely to occur than the other pairs and vice versa.
GLCM_Entropy *	A measure of the amount of information carried by the two-dimensional distribution of pairs of voxel intensities. High values indicates large variability/randomness, low values small variability/randomness.
NGTDM_Busyness *	A measure of the rate of change inversely weighted by the difference in magnitude between the intensity levels.
NGTDM_Coarseness *	A measure of the spatial rate of change between the intensity level of adjacent voxels. Can be interpreted as the size of the primitives in the image: higher values indicate lower spatial change therefore a locally more uniform texture.
NGTDM_Complexity *	A measure of the overall complexity of the image. It is related to the presence of primitive components in the image and the amount of rapid changes in the voxel intensities
*Shape features*	
Elongation	The squared inverse ratio between the largest and the second-largest principal components in the ROI shape. Values close to 0.0 indicate maximal elongation (a line-like, thin object), values close to 1.0 an object with approximately symmetric cross-section (like a square or a circle).
Flatness	The squared inverse ratio between the largest and smallest principal components in the ROI shape. Values close to 0.0 indicate a flat object, values close to 1.0 a sphere-like object.
Sphericity	A measure of the closeness of the ROI shape to that of a sphere. The value ranges between 0.0 and 1.0 with the latter indicating a perfectly spherical ROI.

**Table 3:** Pairwise comparison between average feature values of CT radiomics features in the malignant (“P”) and benign (“N”) groups. Symbol “1” in the “Unit” column indicates a dimensionless feature.

Feature	Unit	N [Mean ± SD]	P [Mean ± SD]	*p*-Value	Alpha	Significant
Minimum	HU	−717.2 ± 104.3	−683.3 ± 150.9	0.0936	0.05/36	No
Maximum	HU	41.8 ± 218.7	64.5 ± 70.1	0.0005	0.05/36	Yes
Mean	HU	−243.0 ± 126.9	−155.2 ± 112.3	0.0001	0.05/36	Yes
StdDev	HU	173.75 ± 34.13	157.95 ± 42.89	0.9754	0.05/36	No
Skewness	1	−0.52 ± 0.62	−1.07 ± 0.74	0.0001	0.05/36	Yes
Kurtosis	1	2.96 ± 1.07	4.14 ± 3.38	0.0088	0.05/36	No
Uniformity	1	0.03 ± 0.01	0.03 ± 0.02	0.2966	0.05/36	No
GLCM_DiffVar	1	455.5 ± 197.6	391.2 ± 193.7	0.9520	0.05/36	No
GLCM_Energy	1	0.012 ± 0.013	0.005 ± 0.003	0.0003	0.05/36	Yes
GLCM_Entropy	1	7.339 ± 1.535	8.432 ± 0.934	<0.0001	0.05/36	Yes
NGTDM_Busyness	1	0.012 ± 0.006	0.012 ± 0.005	0.4791	0.05/36	No
NGTDM_Coarseness	1	0.021 ± 0.015	0.012 ± 0.010	<0.0001	0.05/36	Yes
NGTDM_Complexity	1	70,020.8 ± 41,222.8	76,640.8 ± 48,656.5	0.2455	0.05/36	No
VoxelVolume	mm${}^{3}$	1820.8 ± 2083.8	3647.6 ± 3041.8	<0.0001	0.05/36	Yes
MaxAxialDiameter	mm	16.8 ± 6.3	21.9 ± 6.3	<0.0001	0.05/36	Yes
Sphericity	1	0.769 ± 0.122	0.770 ± 0.077	0.7447	0.05/36	No
Elongation	1	0.742 ± 0.157	0.790 ± 0.116	0.0695	0.05/36	No
Flatness	1	0.560 ± 0.178	0.648 ± 0.127	0.0050	0.05/36	No

**Table 4:** Pairwise comparison between average feature values of PET radiomics features in the malignant (“P”) and benign (“N”) group. Symbol “1” in the “Unit” column indicates a dimensionless feature.

Feature	Unit	N [Mean ± SD]	P [Mean ± SD]	*p*-Value	alpha	Significant
Minimum	SUV	0.9 ± 0.4	1.3 ± 0.5	<0.0001	0.05/36	Yes
Maximum	SUV	3.0 ± 3.2	7.9 ± 3.8	<0.0001	0.05/36	Yes
Mean	SUV	1.6 ± 1.1	3.5 ± 1.3	<0.0001	0.05/36	Yes
StdDev	SUV	0.49 ± 0.68	1.50 ± 0.85	<0.0001	0.05/36	Yes
Skewness	1	0.42 ± 0.42	0.70 ± 0.41	0.0003	0.05/36	Yes
Kurtosis	1	2.65 ± 0.76	2.97 ± 0.80	0.0027	0.05/36	No
Uniformity	1	0.27 ± 0.21	0.06 ± 0.06	<0.0001	0.05/36	Yes
GLCM_DiffVar	1	13.1 ± 34.4	44.4 ± 44.4	<0.0001	0.05/36	Yes
GLCM_Energy	1	0.147 ± 0.171	0.015 ± 0.025	<0.0001	0.05/36	Yes
GLCM_Entropy	1	4.197 ± 2.123	7.222 ± 1.485	<0.0001	0.05/36	Yes
NGTDM_Busyness	1	0.867 ± 1.386	0.164 ± 0.282	<0.0001	0.05/36	Yes
NGTDM_Coarseness	1	0.173 ± 0.171	0.041 ± 0.031	<0.0001	0.05/36	Yes
NGTDM_Complexity	1	1248.0 ± 3700.8	5267.9 ± 6399.6	<0.0001	0.05/36	Yes
VoxelVolume	mm${}^{3}$	2107.7 ± 2409.2	5285.5 ± 3668.3	<0.0001	0.05/36	Yes
MaxAxialDiameter	mm	18.1 ± 5.7	23.4 ± 6.1	<0.0001	0.05/36	Yes
Sphericity	1	0.809 ± 0.110	0.822 ± 0.099	0.2455	0.05/36	No
Elongation	1	0.748 ± 0.117	0.775 ± 0.113	0.1338	0.05/36	No
Flatness	1	0.524 ± 0.213	0.642 ± 0.117	0.0007	0.05/36	Yes

**Table 5:** Estimated performance of the different combination feature sets/classifiers and pairwise differences. Key to symbols: “ACC” = accuracy, “SP” = specificity, “SN” = sensitivity. Values are in %, differences in percentage points. Boldface figures indicate significant differences. For a comparison: accuracy of a random classifier (blind to prior class probabilities) = 50%; with prior class probabilities = 54.4%.

Model	Classifier										
	ClT				KNN				NBGaussian		
	ACC	SN	SP		ACC	SN	SP		ACC	SN	SP
CTb	58.8	67.6	42.2		59.5	65.9	47.5		69.5	83.5	43.3
CTe	62.2	69.6	48.2		70.7	78.7	55.8		74.3	86.9	50.8
PETb	73.4	78.7	63.5		72.5	76.9	64.4		72.2	69.9	76.6
PETe	75.7	82.1	63.6		77.1	81.4	69.1		82.4	87.6	72.6
CTb+PETb	71.2	77.1	60.1		74.4	78.9	66.1		70.6	71.3	69.1
CTe+PETe	72.3	78.9	59.8		73.7	75.7	70.1		80.4	88.5	65.2
CTe-CTb	+ 3.4	+2.0	+ 6.0		+ 11.2	+ 12.8	+ 8.3		+ 4.8	+ 3.4	+ 7.5
PETe-PETb	+ 2.2	+ 3.4	+0.1		+ 4.6	+ 4.5	+ 4.7		+ 10.2	+ 17.7	−3.9
